# Reduction of agalsidase beta infusion time in patients with fabry disease: A case series report and suggested protocol

**DOI:** 10.1016/j.ymgmr.2021.100755

**Published:** 2021-04-15

**Authors:** Aquilino Sánchez-Purificación, Aránzazu Castellano, Belén Gutiérrez, Marta Gálvez, Beatriz Díaz, Tamara Pérez, Francisco Arnalich

**Affiliations:** a Internal Medicine Department, University Hospital La Paz, Madrid, Spain; b Pharmacy Department, University Hospital La Paz, Madrid, Spain

To the Editor,

Fabry disease (FD) is an X-linked rare disorder caused by a deficiency in a lysosomal enzyme. Agalsidase beta (Agal-B) is an enzyme replacement therapy approved for FD patients [1]. The European Summary of Product Characteristics indicates that the initial infusion rate of Agal-B should be no more than 0.25 mg/min until the patient tolerance is stablished, then the infusion rate can be increased gradually [2]. In general practice Agal-B is administered in not less than 90 min. Our service has designed a protocol to substantially reduce the Agal-B infusion times. Patients receive the first 4–8 infusions at 0.25 mg/min and if well tolerated, the infusion time is reduced 30 min in every subsequent infusion until reaching 45 min. 1 g paracetamol is given as premedication. Patients are infused in the hospital and monitored by the nursing staff. This protocol has been applied to our 6 FD patients cohort treated with Agal-B (Fig. 1). In our cohort, only one female presented a dermatological Infusion Associated Reaction (IAR) after the 10^th^ infusion at 45 min. This patient is atopic with a clinical history of several allergies. The IAR was managed with antihistamines and corticosteroid but it repeated in following infusions. Finally, a desensitization protocol was conducted (manuscript in preparation). The only male in our cohort, with a truncating mutation, had a good tolerance. According to the Fabry Registry, males with anti-Agal-B antibodies are more prone to IARs than seronegative patients: 33% vs. 8%, while this difference is 16% vs 10% in women [3]. Although it is necessary to validate this protocol with more classic male patients, Agal-B can be administered in a shorter infusion time than the usual clinical practice. This protocol could help optimize health system resources and improve patient´s quality of life.

**Fig. 1:**
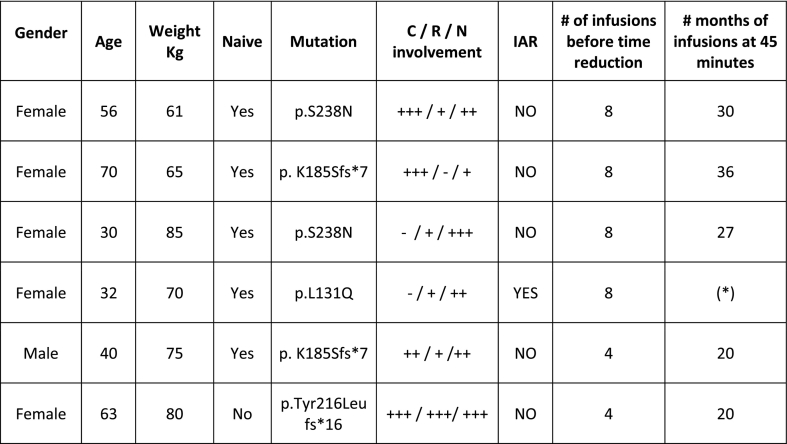
Baseline characteristics of patients receiving agalsidase beta 1.0 mg/kg body weight and infusion information. C: Cardiac; IAR: Infusion Associated Reactions; N: Neurological; R: Renal. (*) This patient had an IAR and is not being infused at 45 minutes at the time, but in longer times.

## Declaration of Competing Interest

Aquilino Sánchez-Purificación has received funding for research, consultancy and lectures from Sanofi Genzyme and Takeda (former Shire HCT).

Francisco Arnalich has received funding for research from Takeda (former Shire HCT).

The rest of authors have no competing interests.
